# High Levels of Leptin and Adipsin Are Associated with Clinical Activity in Early Rheumatoid Arthritis Patients with Overweight and Periodontal Infection

**DOI:** 10.3390/diagnostics13061126

**Published:** 2023-03-16

**Authors:** Consuelo Romero-Sánchez, Juliette De Avila, Alejandro Ramos-Casallas, Lorena Chila-Moreno, Nathaly Andrea Delgadillo, Philippe Chalem-Choueka, César Pacheco-Tena, Juan Manuel Bello-Gualtero, Wilson Bautista-Molano

**Affiliations:** 1 Cellular and Molecular Immunology Group (INMUBO), School of Dentistry, Universidad El Bosque, Av. Carrera 9 No. 131A-02, Bogotá 110121, Colombia; 2 Clinical Immunology Group, Rheumatology and Immunology Department, Hospital Militar Central, Transversal 3 # 49-00, Bogotá 110231, Colombia; 3 Clinical Immunology Group, School of Medicine, Universidad Militar Nueva Granada, Transversal 3 # 49-00, Bogotá 110231, Colombia; 4 Unit of Basic Oral Investigation (UIBO), School of Dentistry, Universidad El Bosque, Av. Carrera 9 No. 131 A-02, Bogotá 110121, Colombia; 5 Fundación Instituto de Reumatología Fernando Chalem, Calle 73 # 20a-27, Bogotá 111211, Colombia; 6 Investigación y Biomedicina De Chihuahua S.C, Calle 16 # 1600 Chihuahua, Chihuahua 31020, Mexico

**Keywords:** adipokines, obesity, periodontitis, rheumatoid arthritis

## Abstract

Adipokines are associated with the pathogenesis of rheumatoid arthritis (RA) and are potential biomarkers of disease activity, periodontitis, and obesity. The aim of this was to establish the association between adipokine profile, RA disease activity, body mass index, and periodontal infection. This study evaluated 51 patients with early-RA and 51 controls including serum rheumatological markers, adipokine levels, detection of *Porphyromonas gingivalis* and serum anti-*Porphyromonas gingivalis* antibodies, clinical and periodontal measurements. Statistical analyses were run with SPSS^®^ V26, with a logistic regression model to confirm associations. The results show high levels of leptin were more frequent in patients (*p* = 0.001) who simultaneously showed a higher frequency of *Porphyromonas gingivalis* (*p* = 0.004). Patients with concomitant presence of *Porphyromonas gingivalis*, high clinical activity score, and overweight were correlated with high levels of leptin (OR, 7.20; 95% CI, 2.68–19.33; *p* = 0.0001) and adipsin (OR, 2.69; 95% CI, 1.00–7.28; *p* = 0.005). The conclusion is that high levels of leptin and adipsin are associated with greater clinical activity in early-RA patients with overweight and periodontal infection, whereby overweight and *Porphyromonas gingivalis* may enhance RA activity. This may represent a pathological mechanism between these conditions, where adipokines seem to have a key role.

## 1. Introduction

Rheumatoid arthritis (RA) is a chronic, inflammatory joint disease of autoimmune nature which can lead to accumulating joint damage and irreversible disability. The disease is complex and involves environmental factors that trigger it in genetically susceptible individuals [1]. Periodontitis mediated by *Porphyoromonas gingivalis* (*P. gingivalis*) infection precedes RA and is a likely factor in the onset and maintenance of the autoimmune response [2].

Some authors recently estimated a 1.49% prevalence in Colombia, through the Community Oriented Program for Control of Rheumatic Diseases strategy [3].

As previously described, patients with early stages of RA (eRA) present a significant incidence of periodontal inflammation (possibly due to periodontal microbiota changes influenced by antirheumatic drugs (DMARDs)) [4] and obesity. The latter is a risk factor related to higher disease activity, functional ability, and health-related quality of life in patients [5]. Based on the evidence regarding the relationship between obesity and periodontitis [6], these two conditions could be linked due to the production of adipokines [7].

Adipokines are a variety of chemical agents (including cytokines and chemokines), which are secreted by adipocytes and non-adipocyte cells (such as fibroblasts, vascular cells, etc.), involved in metabolic and immunity processes [8]. Obesity is a chronic subinflammatory state associated with an altered adipokine profile, with high levels of leptin and reduced adiponectin expression [9].

A similar condition is observed in periodontitis, where this dysregulation correlates with periodontal damage and BMI [10,11]. The proinflammatory profile observed in both conditions could be a related mechanism link. Several studies may support this hypothesis, demonstrating that periodontal ligament cells constitutively secrete adipokines, and external factors can modulate their secretion [12,13].

The association between adipokines and RA development previously described [14] indicates a correlation with radiographic joint damage progression. Leptin seems to be involved in RA, showing positive associations with acute phase markers, disease activity and BMI [15]. In the case of adipsin, serum levels are correlated with BMI, interleukin-6 (IL-6), and erythrocyte sedimentation rate (ESR) in eRA patients [16]. Regarding vaspin, serum levels are associated with the development of clinical manifestations in individuals with serum arthritis biomarkers [17]. The evidence of the potential impact of adipokines as a factor associated with the risk to individuals with developing eRA is limited [15], as most of the studies include patients with established RA [5,14].

Although the mechanisms underlying these associations are not well understood, together, high levels of leptin, the presence of *P. gingivalis*, and overweight may be relevant conditions associated with the development of RA [18]. However, the possibility exists that the adipokine profile is not related to clinical and periodontal conditions in those patients.

Hence, this study aimed to establish the association between adipokine profile, rheumatic activity, BMI, and periodontal infection in eRA patients compared to disease control group.

## 2. Materials and Methods

This study simultaneously recruited controls and patients with a recent diagnosis of RA, according to the ACR/EULAR2010 criteria [19] with fewer than 2 years of disease evolution, in the Department of Rheumatology and Immunology at the Hospital Militar Central, and Fundación Instituto de Reumatología Fernando Chalem, in Bogotá-Colombia from June 2015 to February 2017.

The early-RA (eRA) group was composed of patients between 18 and 65 years old, under only conventional treatment (nonsteroidal anti-inflammatory drugs—NSAIDs, disease-modifying antirheumatic drugs—DMARDs, and/or steroids). The exclusion criteria were ongoing infectious diseases, diagnosis of another autoimmune/autoinflammatory disease, current malignancies, diabetes mellitus, actual orthodontic appliances, antibiotic treatment in the last 3 months, periodontal therapy in the last 6 months, pregnancy, or breastfeeding.

The control group was composed of individuals between 18 and 65 years old, with working or environmental conditions similar to the eRA group. The exclusion criteria for this group were ongoing infectious diseases, diagnosis of another autoimmune/autoinflammatory disease, having familiar history of autoimmune or autoinflammatory diseases, current malignancies, diabetes mellitus, actual orthodontic appliances, antibiotic treatment in the last 3 months, periodontal therapy in the last 6 months, pregnancy, or breastfeeding. Arterial hypertension and overweight were not exclusion variables.

This study was conducted with a nonprobability sampling. Based on the difficulties of recruiting eRA patients in the early stages of the disease and the strict inclusion criteria, the selection was made by convenience. All individuals signed the informed consent form approved by the Hospital Militar Central Institutional Ethics Committee (codes HMC 2016-041 and 2016-099).

### 2.1. Clinical Examination

Rheumatologists measured the level of RA disease activity using the disease activity score 28 (DAS28), the simplified disease activity index (SDAI), the routine assessment of patient index data 3 (RAPID3), and the multidimensional health assessment questionnaire (MDHAQ).

All patients were evaluated by calibrated periodontists, who performed periodontal examinations. A full-mouth examination was performed including selected sites on each permanent tooth, excluding third molars. All patients were evaluated for periodontitis based on the 2017 criteria of the World Workshop on the Classification of Periodontal and Peri-Implant Diseases and Conditions; the stages of periodontitis were determined based on CAL and PD values in interproximal sites [20]. Additionally, periodontal indices, including PD, (inter-examiner intra-class correlation coefficient (IE-ICC), 0.94–0.96) CAL (IE-ICC, 0.92–0.96), bleeding on probing (IE-ICC, 0.88–0.90), plaque index (IE-ICC, 0.94–0.98), and gingival index (IE-ICC, 0.85–0.90), were evaluated in full-mouth examinations including the selected sites.

### 2.2. Adipokine Levels and Serum Markers Measurements

Leptin and vaspin quantifications were calculated using an indirect enzyme-linked immunosorbent assay (ELISA) (Diasource, KAP2281, and MyBioSource, MBS267502 kits, respectively). Quantification of adiponectin, resistin, and adipsin were performed using Luminex xMAP^®^ technology (MILLIPLEX^®^ MAP, HADK1MAG61K03, Merck KGaA, Darmstadt, Germany).

The quantification of IL-6 and CRP were performed using chemiluminescence technology (Immulite 1000, Siemens^®^ Cat. LKP1 No. 10381411, 10286287, Gwynedd, UK) with detectable values of >3.4 pg/mL for IL-6 and high values of >3 mg/L for CRP.

The quantitative measurement of IgG/IgA ACPAs in the serum was performed using a commercially available ELISA kit (Quanta lite^®^ CCP 3.1 IgG/IgA, INNOVA Diagnostics, San Diego, CA, USA) (positive ≥ 20 IU/mL).

The measurement of rheumatoid factor (RF) was calculated using a kinetic turbidimetry technique (Spinreact^®^, 110705, Girona, Spain) (positive ≥ 20 IU/mL), and the ESR using quantitative capillary photometry technology (Alifax Spa^®^, Padova, Italy) (normal value < 20 mm/h).

All the tests were run following the manufacturers’ instructions.

### 2.3. Detection of DNA of P. gingivalis, IgG1 and IgG2 Anti-P. gingivalis Antibodies

DNA of *P. gingivalis* was detected through a quantitative PCR (qPCR) technique. To determine IgG1 and IgG2 antibodies against *P. gingivalis*, indirect ELISA *in-house* assays were performed. These tests were run as described in a previous study [21].

### 2.4. Statistical Analysis

To explore the associations between adipokine levels, rheumatologic activity, BMI, and *P. gingivalis*, an analysis was performed using χ^2^ or Fisher’s exact tests, comparisons were evaluated by Mann–Whitney U tests, and a logistic regression model was made to confirm associations including those variables which showed associations by bivariate analysis. All analyses were conducted using IBM-SPSS software V26 for Windows with a 95% confidence. The effect of sample size on the results was calculated using the matched study power module from Epidat V 4.2.

## 3. Results

In this study, 62 patients with eRA and 62 controls were included. A total of 51 patients and controls matched by age and sex were evaluated.

### 3.1. Sociodemographic Characteristics

The predominant gender was female (80.39%), where BMI score > 25 was higher in patients (50.98%), and smoking conditions were similar between patients and controls (Table 1).

### 3.2. Periodontal Status

The comparison between patients and controls showed that both patients and controls have similar frequency of periodontitis and its severity. However, presence of *P. gingivalis* was more frequent in patients (78.43%), and IgG titers against *P. gingivalis* were higher in controls (Table 2).

### 3.3. Rheumatologic Serum Biomarkers and Disease Activity Scores

Regarding serum biomarkers, 62.74% of eRA patients had levels of RF > 20 IU, 45.09% had levels of ACPA > 20 IU, 56.86% had CRP values higher than 3 mg/L, and 29.41% had prolonged ESR values (>20 mm/h).

The disease activity was measured using the DAS28 score: 62.74% and 58.82% presented a high disease activity score in DAS28-ESR and DAS28-CRP, respectively (>3.2); 58.82% showed elevated disease activity according to SDAI score (>11); 47.05% exhibited high disease severity (>12) according to RAPID-3 score.

### 3.4. Adipokine Profile

To analyze adipokine levels in serum and perform measurements of a continuous variable, cut-off points were necessary to create three equal groups with a canonical stratification based on a percentile 33 (Table 3).

**Figure 1:**
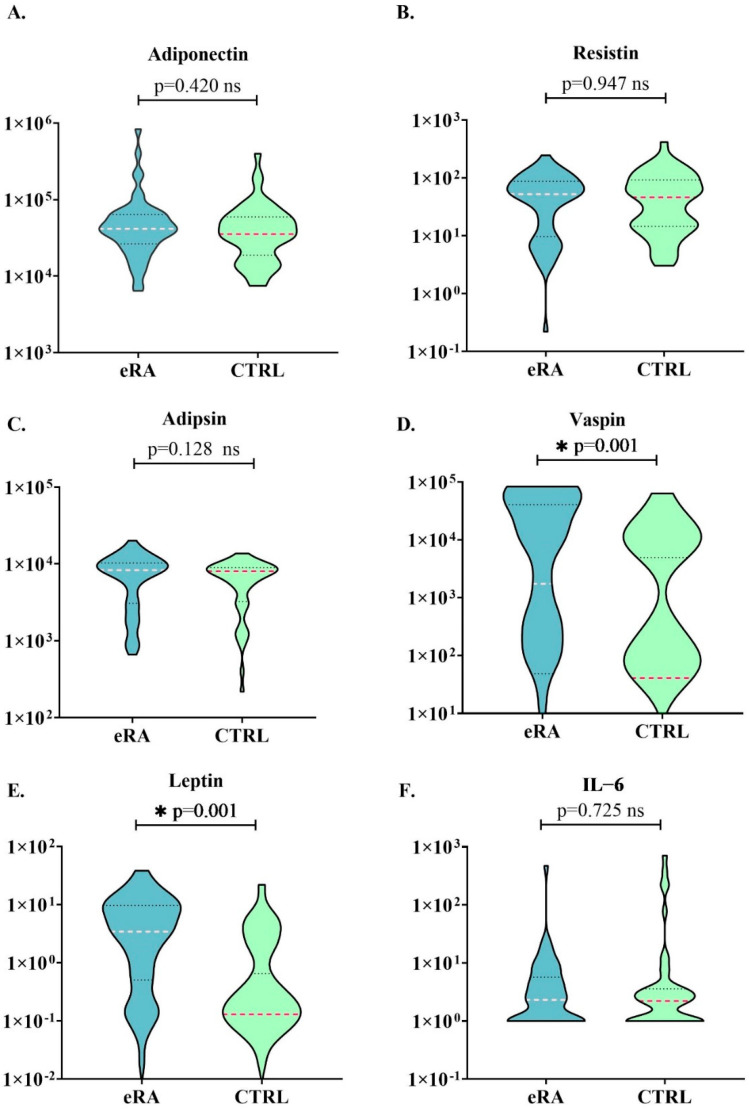
Comparison of adipokine levels in eRA patients and controls. eRA: early–RA group. CTRL: control group. Red/pink line: median of each group; gray lines: quartile 25th and 75th of the graph. * *p* > 0.005.

The frequency of adipokine profile in patients was higher levels of leptin, vaspin, and adipsin, than in controls. Conversely, high levels of adiponectin, resistin, and IL-6 were similar between these groups. A comparison is shown in Figure 1.

### 3.5. Multivariate Analysis

In the multivariate analysis, only leptin and adipsin remained statistically significant. *P. gingivalis* and high levels of leptin and adipsin were simultaneously present in 53.83% and 37.73% of the patients, respectively, with a statistical power of 98.9% for leptin and 94.4% to adipsin.

35.84% of patients were ACPA-positive and had *P. gingivalis*, of which 57.89% had high levels of adipsin. Of these patients, 63.64% manifested high disease activity (DAS28-ESR > 3.2).

Among ACPA-positive patients with *P. gingivalis*, 68.42% and 57.89% had high levels of leptin and adipsin, respectively. Of these patients 62.53% for leptin and 63.64% for adipsin manifested a high disease activity score (DAS28-ESR > 3.2).

In general, these patients were associated with high levels of leptin (OR, 8.22; 95% CI, 2.75–24.50; *p* = 0.001), high levels of adipsin (OR, 3.06; 95% CI, 1.05–8.97; *p* = 0.041), and DAS28-ESR > 3.2 (OR, 2.59; 95% CI, 1.46–4.58; *p* = 0.001).

**Figure 2:**
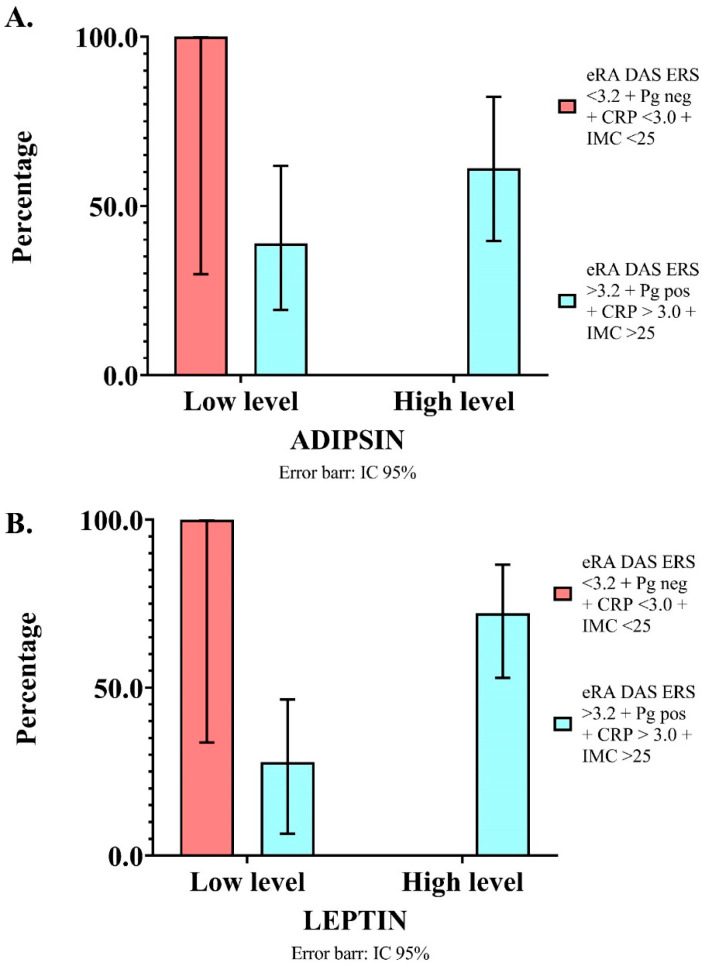
Association between adipokine levels and eRA patients with positive conditions simultaneously: presence of *P. gingivalis*, BMI > 25, CRP > 3.0 mg/L, and DAS28 ESR > 3.2. (**A**) Leptin levels. (**B**) Adipsin levels. *Y* axis: frequency of eRA patients with condition. *X* axis: adipokine levels. BMI: body mass index; CRP: C–reactive protein; *Pg*: *P. gingivalis*.

A total of 22.64% of patients simultaneously had *P. gingivalis*, DAS28-ESR > 3.2, CRP > 3.0 mg/L, and BMI > 25. These patients demonstrated an association with high levels of leptin and adipsin (OR, 7.20; 95% CI, 2.68–19.33; *p* = 0.0001 and OR, 2.69; 95% CI, 1.00–7.28; *p* = 0.005, respectively) (Figure 2). The statistical power for this composite variable was 99.99% both for leptin and adipsin.

Finally, there was no association of adiponectin, resistin, vaspin, or IL-6 with periodontal markers, rheumatology disease activity, and treatment in eRA patients.

## 4. Discussion

RA is a complex systemic autoimmune disease characterized by joint destruction. Environmental and lifestyle factors such as *P. gingivalis*-mediated periodontitis and obesity are associated with a higher risk of developing RA [2,5].

A significant BMI score > 25 was more frequently observed in patients. However, there is a high prevalence of increased BMI scores in healthy people. Kasper et al. reported the increasing rate of obesity in Colombia, correlated with female gender, age, socioeconomic status, and urban residence [22]. With obesity as a high-risk condition for developing comorbid disorders (including RA), these results support that obesity is a public health issue, which will affect the overall quality of life of the population and healthcare costs.

Regarding occupational status, RA patients more frequently reported their occupational status as homemakers, independent workers, or retired. This may be the result of the disability associated with the chronic destructive evolution of RA. According to the follow-up study of Nikiphorou et al., work loss related to RA occurred especially in the first 5 years of RA, but improved over time. The gradual changes in therapies may be one explanation for the differences observed [23]. However, work disability results from a complex interaction between a clinical disease, sociodemographic variables, macroeconomic conditions, and personal factors [24]. Therefore, this condition may imply an integral approach, involving not only the clinical treatment.

As periodontal conditions pose a risk for RA, there was a similar frequency of periodontitis between both groups, with moderate periodontitis the most prevalent condition, with no significant differences. Similar findings have been reported in previous studies, despite the higher detection rate of *P. gingivalis* in patients than in controls [21]. This may be due to the sociocultural and hygienic conditions related to the Colombian population, which could establish the same periodontal conditions in both patients and controls [25]. However, the higher *P. gingivalis* detection in eRA patients has a relevant impact despite no differences in the periodontal status. *P. gingivalis* is a microorganism that can express peptidyl arginine deiminase (PAD), which represents an important pathogenic factor of RA, likely mediating the autoimmune response through citrullination of proteins [2].

Conversely, higher titers in IgG1/2 anti-*P. gingivalis* antibodies were more frequent in controls than in patients. Johansson et al. detected higher anti-*P. gingivalis* antibody titers in RA patients than in controls, a finding suggestive of the role of *P. gingivalis* in the onset of RA [26]. As an extended periodontal pathogen in the general Colombian population [27], we can hypothesize that healthy individuals have an active immunity against this microorganism, thus generating a possible protection mechanism, in contrast to eRA patients because of their systemic compromise and the associated risk factors.

Adipose tissue, through the production of adipokines, is emerging as one of the major drivers of systemic and local inflammation in rheumatic diseases [28]. The higher frequency of patients with BMI > 25 and their positive association with serum leptin levels observed in this study correlates with the findings reported by other authors, where obesity has been associated with higher disease activity and worse quality of life among RA patients [5]. Regarding adipsin levels, the association between BMI > 25 and eRA patients has been reported previously [16]; however, the role of adipsin in the context of obesity and RA is unclear.

In periodontitis, leptin levels increase with a reduction in adiponectin levels, similar to what occurs in obesity [6]. This related mechanism may enhance periodontal disease. Zimmerman et al. found higher leptin levels in obese patients with chronic periodontitis compared to patients with chronic periodontitis and normal weight. The levels of adiponectin were similar in both groups but lower when compared to the control group [11]. Therefore, periodontitis and obesity might be related and can influence each other through adipokine secretion in eRA [7].

The periodontopathic bacteria *P. gingivalis* seems to be involved with adipokines. The presence of *P. gingivalis* was associated with eRA patients with higher levels of leptin and adipsin. The presence of *P. gingivalis* can be a continuous stimulus in the oral cavity, promoting the activation of TLR2 and the synthesis of pro-inflammatory cytokines such as leptin, with a reduction in adiponectin levels [29]. As the immune response is established in the organism, leptin can upregulate TLR2 expression in monocytes [30], where these cells take part in the host defense. The levels of adipsin should also rise, being also produced by monocytes in response to infection, promoting the activation of complement, and favoring the adipsin-dependent pathway to recruit more neutrophils [31,32]. Previous analyses have shown a relationship between the high levels of adiponectin and the absence of *P. gingivalis* [33]. However, this relationship was not found in the current study.

Another additional factor that may mediate the periodontal inflammation and systemic condition in RA patients is oxidative stress. Oxidative stress is an alteration of the balance between the levels of oxidizing agents and those of antioxidants. During normal cellular metabolic processes, free radicals and reactive metabolites are continuously generated. When the rate of oxidant production exceeds the capacity of antioxidant systems to eliminate oxidizing products, oxidative stress is installed, which subsequently leads to cell and tissue damage [34].

It has been noticed that reactive species and antioxidants influence the immune system. Oxidative stress causes disruption in cell signaling, impairs arachidonic acid metabolism, and enhances airway and systemic inflammation [35]. Oxidative stress forms the basis of chronic inflammation, among other diseases [36].

Several oxidative stress biomarkers have been explored in the literature as potentially useful parameters in monitoring the progression of both periodontitis and RA. Biomarkers of oxidative stress (derived from protein damage, lipids, uric acid, and DNA oxidation) were consistently and significantly higher in value in patients with RA compared to systemically healthy individuals, this increase being observed in serum, plasma, urine, synovial fluid, and whole blood [37]. Additionally, Sezer et al. showed that patients with RA and chronic periodontitis presented a high oxidative stress index and prolidase levels [38]. Furthermore, non-surgical periodontal therapy showed an improvement in periodontal conditions and oxidative stress markers in RA patients [39]. While the focus of the current study was to establish the association of adipokines with clinical and periodontal conditions in patients with eRA, it would be interesting to investigate the relationship between adipokines and oxidative stress biomarkers in these patients.

Several rheumatological variables were associated with adipokines. Of the ACPA-positive patients with *P. gingivalis*, 57.89% and 68.42% had higher levels of adipsin and leptin, respectively. More than 60% of them had higher disease activity (DAS28-ESR > 3.2). The higher levels of leptin were related to DAS28-ESR > 3.2 score. Aside from clinical disease activity, inflammatory markers such as CRP seemed to be modulated by adipokines, where higher levels of leptin were related to CRP > 3 mg/L.

Galiutina et al. reported the imbalance of leptin–adiponectin levels. They found that leptin levels in RA patients were 3.2 times higher and that adiponectin levels were 1.7 times lower than those in healthy individuals. In clinical activity, such imbalance was related to the increase in inflammatory markers, such as ESR and CRP. The DAS28 score was 1.6 times higher in individuals with high leptin levels. Thus, leptin levels are increased and are significantly associated not only with disease activity but also with higher levels of CRP [40].

In the case of adipsin, high levels of adipsin were associated with clinical disease (DAS28-ESR > 3.2 score). Additionally, high serum adipsin levels were positively correlated with CRP > 3 mg/L. Although there are no inflammatory properties described, some studies have shown the importance of adipsin in the development of inflammatory arthritis, promoting the migration of neutrophils into joints [32]. Additionally, higher levels of adipsin have been positively correlated with ESR and IL-6 in eRA [16]. Therefore, adipsin seems to be involved in pathological and inflammatory processes including RA, although its role is yet to be elucidated.

Serum levels of vaspin were higher in eRA patients than controls, but this was not related to periodontal and/or rheumatological variables. Maijer et al. described vaspin as a molecule associated with the development of arthritis in ACPA-positive individuals [17]. This may suggest that vaspin could be involved in the development of the initial clinical manifestations.

The serum adipokines leptin, IL-6, and resistin, along with CRP, have been added to a multi-biomarker test as a novel assessment for RA disease activity (MBDA). This test includes the measurement of 12 biomarkers, in which the test score was shown to be positively associated with disease activity and disease relapses in RA patients [41]. However, the “gold standard” assessment for disease activity of RA and the first choice of this study and the cited authors is the use of the DAS28 score instead of MBDA, perhaps due to the technical/economic difficulties of measuring the 12 biomarkers to carry out the MBDA test.

A previous study found a similar relationship between serum adipokines with rheumatological variables of clinical activity and BMI [15]. All of these findings suggest that adipokines play a role in RA pathogenesis and that they may serve as a connection between RA and obesity. However, in this study, we also showed an association between higher leptin and adipsin levels in patients with eRA and the presence of *P. gingivalis*, high index of joint activity, high inflammation markers, and overweight (DAS28-ESR > 3.2, CRP > 3.0 mg/L and BMI > 25). Therefore, adipokines could be the link between obesity, *P. gingivalis*–related periodontitis, and eRA. We found no evidence of research on this topic in the literature reviewed; additional investigations should be encouraged.

While these are important findings, this study presented some limitations. The main limitation of this study is the relatively small number of patients, given the lack of accessibility of the Colombian healthcare system for medical specialist appointments, the difficulties of recruiting patients in the early stages of the disease, and the strict inclusion criteria. In addition, the cross-sectional design of this study does not allow the establishment of the causality of the associations found. Further studies are encouraged, such as prospective follow-up studies with eRA patients, which allow the establishment of the causality of the data related to the adipokine profile and its relationship with rheumatological and periodontal conditions.

## 5. Conclusions

High levels of leptin and adipsin are associated with greater clinical activity in eRA patients with overweight and periodontal infection. These adipokines could be a pathological mechanism whereby overweight and *P. gingivalis* infection might worsen the clinical activity in eRA patients.

## 6. Strengths and Limitations

To the best of the authors’ knowledge, there are few reports evaluating the adipokine profile and its association with periodontal infection and RA activity among eRA patients. In a previous report, the evaluation included a shorter adipokine profile and radiological compromise. The current study measured six different adipokines and evaluated their association with serum rheumatological markers, and clinical and periodontal conditions, which were found to be important associations. This study provides evidence that adipokines may play an essential pathophysiological role in the clinical activity of patients with eRA, overweight, and periodontal infection.

However, the cross-sectional design of this study does not allow the establishment of the causality of the associations found. Furthermore, the main limitation of this study is the relatively small number of patients, given the lack of accessibility of the Colombian healthcare system for medical specialist appointments, the difficulties of recruiting patients in the early stages of the disease, and the strict inclusion criteria applied in this study. It is important to carry out additional studies (especially prospective follow-up studies) on this topic, to find causality associations. Early stages of RA represent an important therapeutic window to use integral approaches to modulate the progression of the disease and improve the quality of life of patients.

A possible bias in this study is the high BMI score in the patients, and the difficulty of recruiting controls who could be matched by sex, age, and BMI score simultaneously. Given that the frequency of overweight is higher in patients with RA, which is supported by the literature, patients and controls were selected under similar environmental and working conditions, and paired by age and sex, not by BMI.

## Figures and Tables

**Table 1:** Sociodemographic characteristics of patients with eRA and controls.

	Controls n = 51		eRA n = 51		*p* Value
	n	%	n	%	
**Age ± SD**	48.20 ± 11.13		48.86 ± 10.86		0.745
**BMI ^†^**	24.52 (21.95–26.34)		25.26 (23.38–28.58)		0.051
**Sex**					
Female	41	80.39	41	80.39	0.521
**BMI > 25**	17	33.33	26	50.98	0.019 *
**Former smoker**	18	35.29	17	33.33	0.552
**Passive smoker**	11	21.56	8	15.68	0.242
**Occupational status**					
Homemaker	12	23.53	19	37.25	0.011 **
Independent worker	0	0.00	3	5.88	
Employee	36	70.59	21	41.18	
Pensioner	2	3.92	6	11.76	
Student	1	1.96	2	3.92	
**Marital status**					
Single	11	21.57	10	19.61	0.140
Widowed	6	11.76	2	3.92	
Common-law marriage	9	17.65	6	11.76	
Separated	5	9.80	3	5.88	
**Education level**					
Elementary school	10	19.61	6	11.76	0.764
High school	11	21.57	15	29.41	
Technician	11	21.57	8	15.69	
College	19	37.25	22	43.14	

BMI: body mass index. ^†^ Data expressed as median (interquartile range). * Statistically significant difference (*p* < 0.05) using Fisher’s test. ** Statistically significant difference (*p* < 0.05) using χ^2^ test.

**Table 2:** Periodontal condition of patients with eRA and controls.

	Controls n = 51		eRA n = 51		*p* Value
	n	%	n	%	
**IgG1 *P. gingivalis* > 1/100 titers**	29	56.86	17	33.33	0.015 *
**IgG1 *P. gingivalis* > 1/400 titers**	18	35.29	8	15.69	0.035 *
**IgG2 *P. gingivalis* > 1/100 titers**	31	60.78	14	27.45	0.003 *
**IgG2 *P. gingivalis* > 1/400 titers**	15	29.41	4	7.84	0.012 *
**IgG1 and IgG2 *P. gingivalis***	37	72.55	20	39.22	0.002 *
***P. gingivalis* presence**	23	45.10	40	78.43	0.003 *
***P. gingivalis* by qPCR > 4 CFU**	22	43.14	31	60.78	0.120
**Periodontal disease diagnostic**					
No compromise	15	29.41	14	27.45	0.575
Periodontitis	36	70.59	37	72.55	
**Severity of periodontal disease**					
No compromise	15	29.41	14	27.45	
Mild	5	9.80	6	11.76	0.411
Moderate	27	52.94	21	41.18	
Severe	4	7.84	10	19.61	

*P. gingivalis*: *Porphyromonas gingivalis*. qPCR: quantitative polymerase chain reaction. CFU: colony-forming unit. * Statistically significant difference (*p* < 0.05) using Fisher’s test.

**Table 3:** Stratification of adipokine levels according to 33rd percentile.

	Tertile 1 Low Levels	Tertile 2 Medium Levels	Tertile 3 High Levels	Median
**Adiponectin (pg/mL)**	0.001–28,303.75	28,303.76–41,753.2	>41,753.2	34,769.60
**Leptin (ng/mL)**	0.001–0.15	0.16–1.67	>1.67	0.18
**IL-6 (pg/mL)**	0.001–1.0	1.1–2.29	>2.29	1.0
**Resistin (pg/mL)**	0.001–16.83	16.84–65.69	>65.69	49.34
**Adipsin (pg/mL)**	0.001–3076.9	3076.9–8729.5	>8729.58	7728.36
**Vaspin (pg/mL)**	0.001–68.80	68.81–1273.1	>1273.19	87.50

Low level: percentile < 33rd. Medium level: percentile 33–66th High level: percentile > 66th. Median: 50th percentile.

## Data Availability

The data used to support the findings of this study are available from the corresponding authors upon request.
